# Research on the Equivalent Span of Hybrid Girder Bridges

**DOI:** 10.3390/ma18061278

**Published:** 2025-03-13

**Authors:** Bing Shangguan, Feng Wang, Qingtian Su, Fawas O. Matanmi, Jun Xu

**Affiliations:** 1 Department of Bridge Engineering, Tongji University, 1239 Siping Road, Shanghai 200092, China; shangguanbing@tongji.edu.cn (B.S.); 2393186@tongji.edu.cn (F.O.M.); 2180025@tongji.edu.cn (J.X.); 2 Guangdong Yejian Construction Drawing Review Center Co., Ltd., Guangzhou 510062, China; 3 Communications Comprehensive Planning and Design Institute Co., Ltd., Beijing 100024, China; wangfengfendou@126.com; 4 CCCC Highway Consultants Co., Ltd., Beijing 100010, China

**Keywords:** hybrid girder component, simplified analytical model, Leq method, equivalent span increase coefficient, preliminary design, span arrangement, ultimate span

## Abstract

Hybrid girder bridges achieve significant improvements in spanning capacity by utilizing lightweight and high-strength materials in the midspan beam segments. To quantitatively describe the enhancement in spanning capacity, this study introduces a simplified analytical model for hybrid girder components, avoiding complex factors, such as span ratio and boundary conditions, typically encountered in previous system-level analyses. The Leq method is proposed based on this new model, utilizing classical beam theory model to calculate and compare hybrid girder components with both uniform and variable cross-sections. The equivalent span increase coefficient, κ, is introduced for the first time, and a simplified formula for its calculation is derived. The calculation errors are kept within 8%, which meets the requirements for preliminary design. Validated through engineering practice, the formula is concise and reveals that κ is solely related to the hybrid ratio, μ, and the linear load ratio, γ. This method provides valuable guidance for the conceptual design and ultimate span prediction of hybrid girder bridges.

## 1. Introduction

In the 1990s, as large-span prestressed concrete girder bridges began experiencing issues such as web cracking and progressive deflection to varying degrees, hybrid girder bridges emerged as a solution. By using lightweight and high-strength materials in the main girder at the midspan to replace concrete girders, hybrid girder bridges reduced self-weight, allowing for larger bridge spans and mitigating girder deterioration to some extent. Notable examples, such as the Stolma Bridge [1] and the Shibanpo Bridge in Chongqing [2], successively broke the world record for the span of solid-web girder bridges, demonstrating the advantages of hybrid girder bridges.

A hybrid girder bridge is characterized by main girders made of different materials that are effectively connected along the longitudinal direction of the bridge to function as a unified structure under load. This concept is quite similar to plant grafting [3,4,5,6], in which the rootstock, which is connected to the soil, supports the scion, forming a grafted plant. The grafted plant combines the advantages of both the rootstock and the scion, resulting in superior overall performance. Inspired by plant grafting, this paper names the components of hybrid girder bridges as follows: In a hybrid girder bridge, the girder segments not connected to the main foundation of the bridge are called scion beams, while the girder segments connected to the main foundation and bearing the load from the scion beams are referred to as rootstock beams.

**Figure 1:**
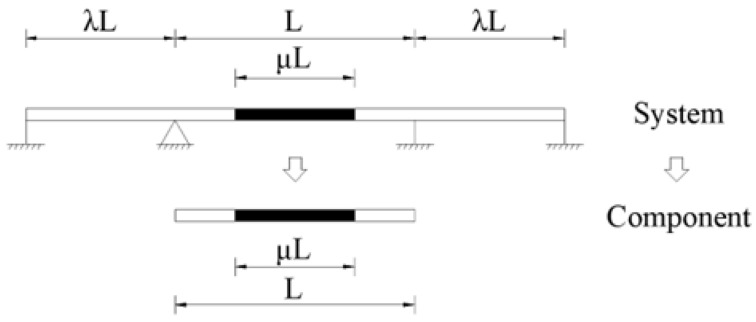
Hybrid girder bridge system and hybrid girder component.

Current research on hybrid girder bridges primarily focuses on the mechanical performance of joints [7,8,9,10,11] and system-level analysis. Most studies [12,13,14,15,16,17,18] of the latter category aim to determine the optimal span ratio between side and middle spans, *λ*, as well as the optimal hybrid ratio, μ, for three-span hybrid girder bridge systems. However, as the simplified mechanical models for three-span hybrid girder bridges become increasingly refined, the analytical solutions also grow more complex, which, ironically, hinders their practical application in engineering projects. Structural systems are composed of components, and only by conducting in-depth research on the fundamental components can the force characteristics of the entire system be better understood [19]. Given that hybrid girder bridges are primarily bending-dominated structural systems, the study of the bending characteristics of hybrid girder components is particularly significant, as illustrated in Figure 1.

In 2010, Man-Chung Tang [20] proposed the concept of an equivalent span for the Shibanpo Bridge. However, his simplified model, which divided a uniform cross-section beam into three equal parts, could not account for the effects of important factors such as the hybrid ratio, load ratio, stiffness ratio, and beam height on the equivalent span, thereby presenting certain limitations. Drawing on Tang’s concept of equivalent span, this study introduces the Leq Method for analyzing the bending characteristics of hybrid girder components, focusing on the relationship between hybrid girder components and rootstock beam components. For brevity, terms such as “hybrid girder components”, “rootstock beam components”, and “scion beam components” will be abbreviated as “hybrid girder”, “rootstock beam”, and “scion beam”, respectively, in contexts such as model development and formula derivation.

The equivalent span refers to the span of a rootstock beam component that produces the same internal force at the critical section as the hybrid girder component. The ratio of the two spans is defined as the equivalent span increase coefficient, κ, which is influenced by factors such as the hybrid ratio, μ, load ratio, γ, stiffness ratio, β, and beam height, *h*. As the core parameter in equivalent span research, κ not only reflects the mechanical advantages of hybrid girder components but also serves as a key factor in the system design of hybrid girder bridges, providing valuable guidance for engineering practice. This study aims to propose a simplified formula for κ and to apply it to guide the conceptual design of hybrid girder bridges.

## 2. Research Assumptions

The bending model of hybrid girder components is entirely based on elastic linear analysis. Hybrid girder bridges have a low height-to-span ratio, making classical beam theory (Euler–Bernoulli beam) sufficient to meet the accuracy requirements of engineering applications. The computational simplicity of classical beam theory also facilitates the derivation of intuitive analytical solutions. Therefore, in the bending model of the hybrid girder components, both the rootstock beam components and hybrid girder components are assumed to be classical beam elements. This model adheres to the plane section assumption, neglecting the effects of rotational inertia, shear deformation, geometric nonlinearity, material nonlinearity, and temperature. To further derive results with practical engineering significance, the following additional assumptions and conventions are established:

(1) Load. The load in the bending model of hybrid girder components is represented as a line load perpendicular to the hybrid girder. The intensity of the line load is related to factors such as the material’s unit weight and quantity.
(2) Cross-sectional properties. The bending model of hybrid girder components is classified into two types based on cross-sectional properties: uniform cross-section beams and variable cross-section beams. For variable cross-section beams, the beam height varies according to a power function with an exponent *α*.
(3) Boundary Conditions. The boundary conditions of the bending model for hybrid girder components are classified into two types: fixed at both ends and hinged at both ends. Fixed at both ends (referred to as the fixed-end beam model): This model has complete boundary constraints, capable of providing vertical reactions, horizontal reactions, and fixed-end moments. Additionally, if initial moments are applied at the fixed ends, the model can fully simulate the bending behavior of hybrid girder components. However, the fixed-end beam model involves numerous parameters, making it challenging to derive explicit analytical solutions during theoretical derivations. Hinged at both ends (referred to as the simply supported beam model): This model provides boundary constraints that offer only vertical and horizontal reactions but no fixed-end moments. Despite this limitation, the maximum bending moment, *M_max_*, in a simply supported beam model is equivalent to the difference, *ΔM*, between the maximum and minimum bending moments in a fixed-end beam model. Since *ΔM* and *M_max_* have analogous significance in evaluating the spanning capacity of girder bridges, the simply supported beam model holds value as an alternative. Its primary advantage lies in having fewer parameters, which simplifies theoretical analysis and allows for easier derivation of explicit analytical solutions.
(4) Equivalent internal force terms. When the maximum bending moment of the rootstock beam component is set equal to the maximum bending moment of the hybrid girder component as the equivalence principle, the resulting equivalent span increase coefficient is referred to as the equivalent bending moment span increase coefficient $\kappa_{M}$. When the maximum shear force of the rootstock beam component is set equal to the maximum shear force of the hybrid girder component as the equivalence principle, the resulting equivalent span increase coefficient is referred to as the equivalent shear force span increase coefficient $\kappa_{V}$.

## 3. Study on the Equivalent Span of Uniform Cross-Section Hybrid Girders

### 3.1. κ Based on the Fixed-End Beam Model

**Figure 2:**
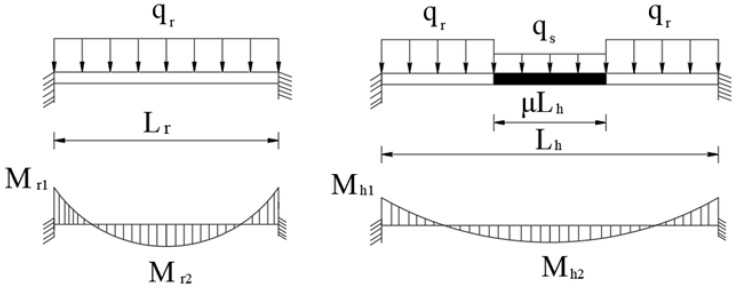
Fixed-end beam model and bending moment diagram of a uniform cross-section hybrid girder.

When analyzing the coefficient $\kappa_{M}$ of uniform cross-section hybrid girder components using the fixed-end beam model, the mechanical models and bending moment diagrams of the rootstock beam and hybrid girder are illustrated in Figure 2.

For a fixed-end beam under load, the maximum negative bending moment occurs at the fixed ends, while the maximum positive bending moment occurs at mid-span. Based on the fundamental principles of structural mechanics [21,22], the fixed-end negative bending moments of the rootstock beam and hybrid girder are calculated using Equation (1), and the mid-span positive bending moments of the rootstock beam and hybrid girder are calculated using Equation (2). The derivation of the aforementioned formula is provided in detail in Appendix A.

(1) $$\left\{\begin{array}{c} M_{r1}=-\frac{1}{12}q_{r}L_{r}^{2}\\ M_{h1}=-\frac{3\beta \gamma \mu {\left(1-\mu \right)}^{2}+2\beta {\left(1-\mu \right)}^{3}+\left(6-4\mu \right)\gamma \mu^{2}+3\mu {\left(1-\mu \right)}^{2}}{24\left[\beta \left(1-\mu \right)+\mu \right]}q_{r}L_{h}^{2} \end{array}\right.$$

(2) $$\left\{\begin{array}{c} M_{r2}=\frac{1}{24}q_{r}L_{r}^{2}\\ M_{h2}=\frac{3\beta \gamma \mu \left(1-\mu \right)+\beta {\left(1-\mu \right)}^{3}+\gamma \mu^{3}}{24\left[\beta \left(1-\mu \right)+\mu \right]}q_{r}L_{h}^{2} \end{array}\right.$$

where β = (*E_s_I_s_*)/(*E_r_I_r_*) and γ = *q_s_/q_r_*.

When *M_r_*_1_ = *M_h_*_1_, the corresponding *L_r_* and *L_h_* constitute the equivalent negative bending moment span. The span ratio between the hybrid girder and the rootstock beam is defined as the equivalent negative bending moment span increase coefficient $\kappa_{M}^{-}$, given by Equation (3):

(3) $$\kappa_{M}^{-}=\frac{L_{h}}{L_{r}}=\sqrt{\frac{2\left[\beta \left(1-\mu \right)+\mu \right]}{3\beta \gamma \mu {\left(1-\mu \right)}^{2}+2\beta {\left(1-\mu \right)}^{3}+\left(6-4\mu \right)\gamma \mu^{2}+3\mu {\left(1-\mu \right)}^{2}}}$$

Similarly, when *M_r_*_2_ = *M_h_*_2_, the equivalent positive bending moment span increase coefficient $\kappa_{M}^{+}$ is given by Equation (4):

(4) $$\kappa_{M}^{+}=\frac{L_{h}}{L_{r}}=\sqrt{\frac{\left[\beta \left(1-\mu \right)+\mu \right]}{3\beta \gamma \mu \left(1-\mu \right)+\beta {\left(1-\mu \right)}^{3}+\gamma \mu^{3}}}$$

When analyzing the coefficient $\kappa_{V}$ of uniform cross-section hybrid girder components based on the fixed-end beam model, the maximum shear force of both the rootstock beam and the hybrid girder occurs at the fixed end.

(5) $$\left\{\begin{array}{c} V_{r}=\frac{1}{2}q_{r}L_{r}\\ V_{h}=\frac{1}{2}q_{r}L_{h}\left(1-\mu \right)+\frac{1}{2}q_{s}L_{h}\mu \end{array}\right.$$

When *V_r_* = *V_h_*, the coefficient $\kappa_{V}$ is given by Equation (5).

(6) $$\kappa_{V}=\frac{L_{h}}{L_{r}}=\frac{1}{\left(1-\mu \right)+\mu \gamma }$$

### 3.2. κ Based on Simply Supported Beam Model

**Figure 3:**
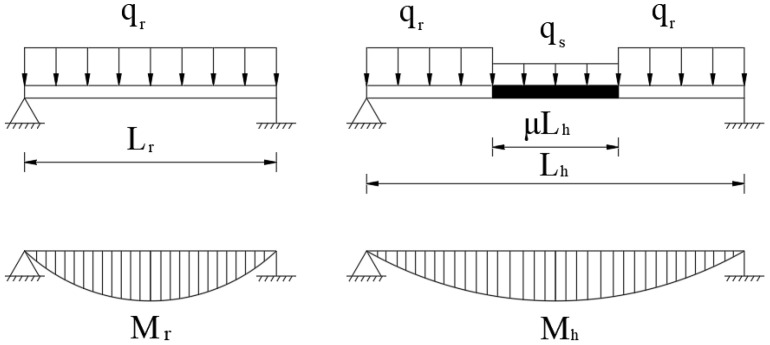
Simply supported beam model and bending moment diagram of a uniform cross-section hybrid girder.

When analyzing the coefficient $\kappa_{M}$ of uniform cross-section hybrid girder components using the simply supported beam model, the mechanical models and bending moment diagrams of the rootstock beam and hybrid girder are illustrated in Figure 3.

For a simply supported beam under load, the maximum bending moment occurs at mid-span. The mid-span positive bending moments of the rootstock beam and hybrid girder are calculated using Equation (7):

(7) $$\left\{\begin{array}{c} M_{r}=\frac{1}{8}q_{r}L_{r}^{2}\\ M_{h}=\frac{1}{8}q_{r}L_{h}^{2}{\left(1-\mu \right)}^{2}+\frac{1}{8}q_{s}L_{h}^{2}\left[\mu \left(2-\mu \right)\right] \end{array}\right.$$

When *M_r_* = *M_h_*, the coefficient $\kappa_{M}$ is given by Equation (8):

(8) $$\kappa_{M}=\frac{L_{h}}{L_{r}}=\sqrt{\frac{1}{{\left(1-\mu \right)}^{2}+\left(2\mu -\mu^{2}\right)\gamma }}$$

When analyzing the coefficient $\kappa_{V}$ of uniform cross-section hybrid girder components using the simply supported beam model, the maximum shear forces of the rootstock beam and hybrid girder occur at the supports. When *V_r_* = *V_h_*, the coefficient $\kappa_{V}$ is also given by Equation (6).

### 3.3. Comparison of κ

The two models above yield four κ values, namely, $\kappa_{M}^{-}$, $\kappa_{M}$, $\kappa_{M}^{+}$ and $\kappa_{V}$. A comparative analysis is as follows:

Under the condition of a fixed γ, boundary point analysis is conducted with $\kappa =f\left(\mu \right)$.

When μ=0, $\kappa_{M}^{-}=\kappa_{M}=\kappa_{M}^{+}=\kappa_{V}=1$;

When μ=1, $\kappa_{M}^{-}=\kappa_{M}=\kappa_{M}^{+}=\frac{1}{\sqrt{\gamma }}$ < $\kappa_{V}=\frac{1}{\gamma }$;

As μ approaches 1, $\kappa_{V}$ becomes significantly larger than the other three coefficients, and Equation (9) also proves that $\kappa_{V}$ is consistently greater than $\kappa_{M}$. Therefore, shear force is not the controlling factor, and $\kappa_{V}$ is not suitable as the optimal κ.

(9) $${\left(\kappa_{V}\right)}^{2}=\frac{1}{{\left(1-\mu \right)}^{2}+\left(2\mu -2\mu^{2}\right)\gamma +\mu^{2}\gamma^{2}}>{\left(\kappa_{M}\right)}^{2}$$

The comparison among $\kappa_{M}^{-}$, $\kappa_{M}$, and $\kappa_{M}^{+}$ is quite challenging, as repeated verification shows that there is no absolute fixed relationship among the three. Below, the differences between $\kappa_{M}^{-}$, $\kappa_{M}$, and $\kappa_{M}^{+}$ are analyzed using two examples of hybrid girders commonly used today: steel-concrete hybrid girders and lightweight concrete hybrid girders.

**Figure 4:**
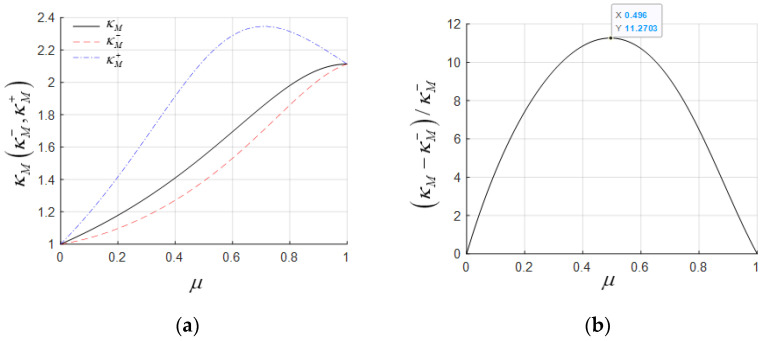
Coefficient comparison analysis of steel–concrete hybrid girders: (**a**) equivalent moment span increase coefficient; (**b**) relative difference between $\kappa_{M}$ and $\kappa_{M}^{-}$.

For a three-span steel-concrete hybrid girder, the steel box girder has a load of 110.29 kN/m, a bending moment of inertia of 2.889 m^4^, and a modulus of elasticity of 2.06 × 10^5^ Mpa. The concrete box girder has a load of 491.78 kN/m, a bending moment of inertia of 37.711 m^4^, and a modulus of elasticity of 3.55 × 10^4^ Mpa. This gives γ = 0.224 and β = 0.444. The calculation results for $\kappa_{M}^{-}$, $\kappa_{M}$, and $\kappa_{M}^{+}$ are illustrated in Figure 4a, where $\kappa_{M}^{-}$ < $\kappa_{M}$ < $\kappa_{M}^{+}$. The $\kappa_{M}^{+}$ value is relatively large, and its curve exhibits abnormal non-monotonic growth, leading to failure. In contrast, the curves for $\kappa_{M}^{-}$, $\kappa_{M}$ are normal and very close to each other, with a maximum error of 11.27%, as shown in Figure 4b.

**Figure 5:**
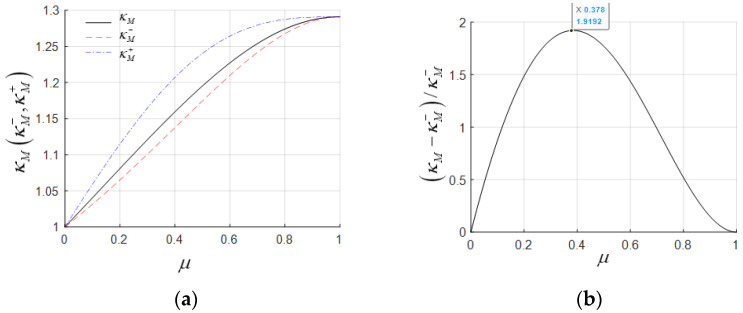
Coefficient comparison analysis of lightweight concrete hybrid girders: (**a**) equivalent moment span increase coefficient; (**b**) relative difference between $\kappa_{M}$ and $\kappa_{M}^{-}$.

For a lightweight concrete hybrid girder with γ = 0.6 and β = 1, the calculation results for $\kappa_{M}^{-}$, $\kappa_{M}$, and $\kappa_{M}^{+}$ are illustrated in Figure 5a. The relationship remains $\kappa_{M}^{-}$ < $\kappa_{M}$< $\kappa_{M}^{+}$. The curves for $\kappa_{M}^{-}$ and $\kappa_{M}$ are still very close, with a maximum error of 13.99%, as shown in Figure 5b.

In general, negative bending moments play a controlling role in hybrid girder design, making $\kappa_{M}^{-}$ a more accurate choice as the equivalent span length increase factor. From the two examples above, it can be observed that $\kappa_{M}^{+}$ is larger than $\kappa_{M}^{-}$ and deviates significantly, making it unsuitable as the optimal κ. Meanwhile, $\kappa_{M}$ and $\kappa_{M}^{-}$ show consistent variation patterns and have minimal numerical differences, each with its own advantages. The expression for $\kappa_{M}$ (Equation (8)) does not depend on β and combines both computational accuracy and a uniquely simple and clear formulation, making it well-suited for use in the conceptual design phase.

### 3.4. Characteristics of $\kappa_{M}$

**Figure 6:**
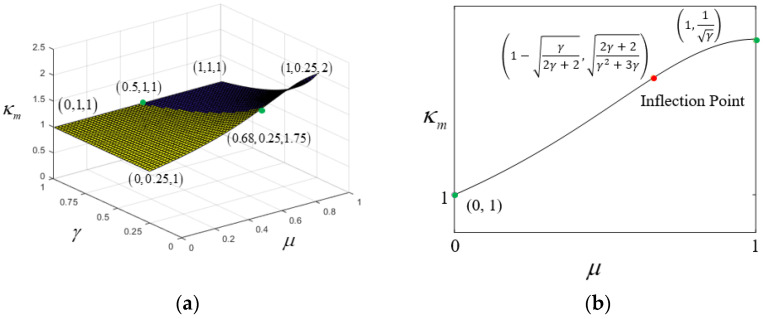

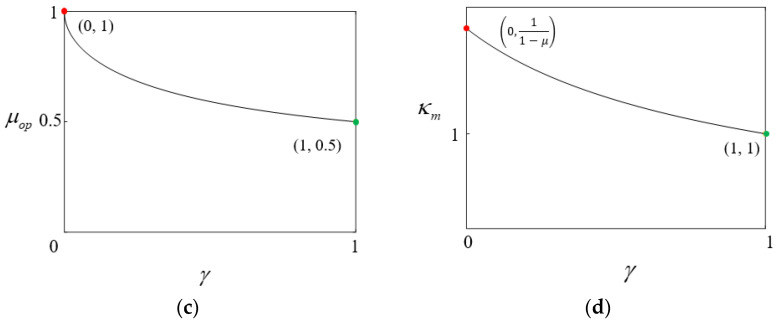
Characteristics of $\kappa_{M}$ for uniform cross-section hybrid girders (**a**) $\kappa_{M}(\mu ,\gamma )$; (**b**) $\kappa_{M}\left(\mu \right)$; (**c**) $\mu_{op}$; (**d**) $\kappa_{M}(\gamma )$.

$\kappa_{M}$ is a bivariate function of μ and γ, as expressed in Equation (8). A 3D surface plot of $\kappa_{M}$ (μ∈ (0, 1), γ∈ (0.25, 1)) has been created, resembling a surface formed by lifting one corner of a rectangular plane, as shown in Figure 6a. Based on mathematical analysis of $\kappa_{M}$ (detailed in Appendix B), its characteristics are as follows:

1. When parameter γ is constant, within the range μ∈ (0, 1), $\kappa_{M}$ (μ) forms a monotonic curve that is initially concave and later convex, with endpoints at (0, 1), (1, $\frac{1}{\sqrt{\gamma }}$) and an inflection point at $\left(1-\sqrt{\frac{\gamma }{2\gamma +2}},\sqrt{\frac{2\gamma +2}{\gamma^{2}+3\gamma }}\right)$, as shown in Figure 6b.

The curve indicates that when γ is constant, the larger μ is, the larger $\gamma_{M}$ becomes. The rate of increase in $\kappa_{M}$ peaks at the inflection point and then gradually decreases. Therefore, the horizontal coordinate of the inflection point corresponds to the theoretically optimal hybrid ratio $\mu_{op}$, as expressed in the following formula:

(10) $$\mu_{op}=1-\sqrt{\frac{\gamma }{2\left(1+\gamma \right)}}$$

Equation (10) indicates that as γ changes from 1 to 0, the optimal hybrid ratio varies from 0.5 to 1, as shown in Figure 6c.

2. When parameter μ is constant, within the range γ∈ (0, 1), $\kappa_{M}(\gamma )$ forms a concave curve with monotonic increase and decrease, with endpoints at $\left(0,\frac{1}{1-\mu }\right)$ and (1, 1), as shown in Figure 6d.

## 4. Study on Equivalent Span Increase Coefficient of Variable Cross-Section Hybrid Girders

The parameters of the variable cross-section hybrid girder model increase, making it difficult to derive a mechanical analytical solution for $\kappa_{M}$ using the fixed-end beam model. The derivation in the previous section has already demonstrated that using the simply supported beam model to solve for $\kappa_{M}$ yields consistent trends with the fixed-end beam model, with acceptable differences. Therefore, this study employs only the simply supported beam model to analyze $\kappa_{M}$ for variable cross-section hybrid girders, omitting shear force-based coefficient $\kappa_{V}$ for the same reasons as in the previous section.

**Figure 7:**
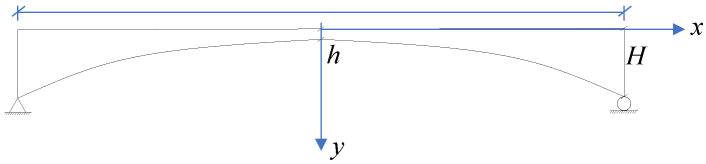
Geometric schematic of the variable cross-section hybrid girder based on the simply supported beam model.

The geometric shape of the simply supported variable cross-section girder changes according to a power function, as illustrated in Figure 7. The girder height and load of the variable cross-section hybrid girder are given in Equations (11) and (12):

(11) $$h\left(x\right)=h+\frac{H-h}{{\left(0.5L\right)}^{\alpha }}x^{\alpha }$$

(12) $$q\left(x\right)=\beta_{1}h\left(x\right)+\beta_{2}=\frac{\beta_{1}\left(H-h\right)}{{\left(0.5L\right)}^{\alpha }}x^{\alpha }+\left(\beta_{1}h+\beta_{2}\right)$$

**Figure 8:**
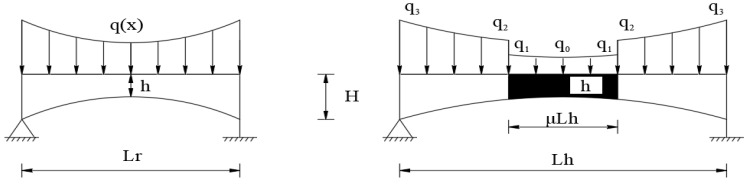
Simply supported beam model and load diagram of the variable cross-section hybrid girder.

The corresponding load diagram for the variable cross-section hybrid girder is shown in Figure 8.

Based on the load diagram of the hybrid girder, the following load intensity relationships are expressed in Equation (13):

(13) $$q\left(x\right)=\left\{\begin{array}{c} \beta_{1s}\left(h+\frac{H-h}{{\left(\frac{L_{h}}{2}\right)}^{\alpha }}x^{\alpha }\right)+\beta_{2s}\;\;\;\;x\in \left(0,\mu L_{h}/2\right)\\ \beta_{1r}\left(h+\frac{H-h}{{\left(\frac{L_{h}}{2}\right)}^{\alpha }}x^{\alpha }\right)+\beta_{2r}\;\;\;\;x\in \left(\mu L_{h}/2,L_{h}/2\right) \end{array}\right.$$

For a simply supported beam under load, the maximum bending moment occurs at mid-span. The mid-span positive bending moments for both the rootstock beam and the hybrid girder are calculated using Equations (14) and (15):

(14) $$\begin{array}{cc} M_{r} & =\int_{0}^{L_{r}/2}q_{r}\left(x\right)\left(\frac{L_{r}}{2}-x\right)dx=\int_{0}^{L_{r}/2}\left(\frac{\beta_{1r}\left(H-h\right)}{{\left(0.5L_{r}\right)}^{\alpha }}x^{\alpha }+\left(\beta_{1r}h+\beta_{2r}\right)\right)\left(\frac{L_{r}}{2}-x\right)dx\\ & =\left(\frac{\left(\beta_{1r}h+\beta_{2r}\right)}{8}+\frac{\left(H-h\right)\beta_{1r}}{4\left(\alpha +1\right)\left(\alpha +2\right)}\right)L_{r}^{2} \end{array}$$

(15) $$\begin{array}{cc} M_{h} & =\int_{0}^{\mu L_{h}/2}q_{s}\left(x\right)\left(\frac{L_{h}}{2}-x\right)dx+\int_{\mu L_{h}/2}^{L_{h}/2}q_{r}\left(x\right)\left(\frac{L_{h}}{2}-x\right)dx\\ & =\int_{0}^{\mu L_{h}/2}\left(\frac{L_{h}}{2}-x\right)\left(\beta_{1s}\left(h_{h}+\frac{H-h}{{\left(L_{h}/2\right)}^{\alpha }}x^{\alpha }\right)+\beta_{2s}\right)dx+\int_{\mu L_{h}/2}^{L_{h}/2}\left(\frac{L_{h}}{2}-x\right)\left(\beta_{1r}\left(h_{h}+\frac{H-h}{{\left(L_{h}/2\right)}^{\alpha }}x^{\alpha }\right)+\beta_{2r}\right)dx\\ & =\left(\frac{\left(2\mu -\mu^{2}\right)\left(\beta_{1s}h+\beta_{2s}\right)}{8}+\frac{\left(H-h\right)\beta_{1s}}{4}\left(\frac{\mu^{\alpha +1}}{\alpha +1}-\frac{\mu^{\alpha +2}}{\alpha +2}\right)\right)L_{h}^{2}+\left(\frac{{\left(1-\mu \right)}^{2}\left(\beta_{1r}h+\beta_{2}\right)}{8}+\frac{\left(H-h\right)\beta_{1r}}{4}\left(\frac{1-\mu^{\alpha +1}}{\alpha +1}-\frac{1-\mu^{\alpha +2}}{\alpha +2}\right)\right)L_{h}^{2} \end{array}$$

When the maximum positive bending moments of the rootstock beam and the hybrid girder (*M_r_* = *M_h_*) are equal, $\kappa_{M}$ can be derived by Equation (16).

(16) $$\kappa_{M}=\sqrt{\frac{\left(\beta_{1r}h+\beta_{2r}\right)+2\left(H-h\right)\beta_{1r}\frac{1}{\left(\alpha +1\right)\left(\alpha +2\right)}}{{\left(1-\mu \right)}^{2}\left(\beta_{1r}h+\beta_{2r}\right)+\left(2\mu -\mu^{2}\right)\left(\beta_{1s}h+\beta_{2s}\right)+2\left(H-h\right)\left(\beta_{1r}\left(\frac{1-\mu^{\alpha +1}}{\alpha +1}-\frac{1-\mu^{\alpha +2}}{\alpha +2}\right)+\beta_{1s}\left(\frac{\mu^{\alpha +1}}{\alpha +1}-\frac{\mu^{\alpha +2}}{\alpha +2}\right)\right)}}$$

## 5. Engineering Case Comparison of $\kappa_{M}$ Between Uniform and Variable Cross-Sections

To compare the accuracy and differences of $\kappa_{M}$ between uniform and variable cross-sections, five large-span hybrid girder bridges, both domestic and international, were selected: Stolma Bridge, Shibanpo Bridge, Oujiang Bridge, Anhai Bay Bridge, and Taoer River Bridge. By consulting relevant technical reports [1,23,24,25,26], the equivalent internal force span amplification factors ${\kappa_{M}}_{1}$, ${\kappa_{M}}_{2}$ and ${\kappa_{M}}_{3}$ were calculated: ${\kappa_{M}}_{1}$ was calculated using Equation (16); and ${\kappa_{M}}_{2}$ and ${\kappa_{M}}_{3}$ were calculated using Equation (8). For ${\kappa_{M}}_{2}$, the parameter γ was determined based on the actual weight ratio of the bridges. For ${\kappa_{M}}_{3}$, the parameter γ was derived based on empirical values. The calculation results are presented in Table 1.

The calculated value using the variable cross-section formula, ${\kappa_{M}}_{1}$, is considered the benchmark, as it provides the smallest error. When compared to the results from the uniform cross-section formula, ${\kappa_{M}}_{2}$ and $\kappa_{M3}$, the error rate ranges from a maximum of 7.4% to a minimum of 3.2%, which meets the accuracy requirements for conceptual design and scheme design. Among the three coefficients, $\kappa_{M3}$ is called the $\kappa_{M}$ estimation formula. It only depends on μ and γ, and γ is based on empirical values without the need for calculation. This formula combines computational accuracy with simplicity and clarity, offering unique advantages and broad application value in scheme design.

## 6. Applications

With the $\kappa_{M}$ estimation formula, it becomes easier to implement the span design and ultimate span prediction for hybrid girder bridges.

### 6.1. Span Arrangement Design

Based on the $\kappa_{M}$ estimation formula, the equivalent span *L_eq_* = *L*/$\kappa_{M}$ of the hybrid girder can be calculated. By combining the commonly used edge-to-midspan ratios for continuous beams (or continuous rigid frames) of 0.5~0.68, the appropriate span of the edge spans can be easily arranged.

Table 2 presents the verification results from 14 worldwide typical hybrid girder bridges.

Among the fourteen hybrid girder bridges, only two bridges have side spans that fall outside the reasonable span range due to pier position constraints. This demonstrates the high rationality and practicality of the equivalent internal force span method (Leq Method) in hybrid girder bridge span layout design. Similarly, if the span layout of a hybrid girder bridge is fixed, the equivalent internal force span method (Leq Method) can be used to quickly calculate the reasonable value of μ.

### 6.2. Limit Span Prediction

According to engineering practice, the limit span of hybrid girders is related to factors such as the hybrid ratio, the load ratio, and the construction technology of the rootstock girder and scion girder. Among these, μ is an adjustable factor. As shown in Figure 6b, when μ changes from 0 to 1, $\kappa_{M}$ changes from 1 to 1$/\sqrt{\gamma }$, and *L_h_* changes from *L_r_* to *L_r_*$/\sqrt{\gamma }$. When *L_r_*$/\sqrt{\gamma }$ < *L_s_*, the span advantage of the hybrid girder is lost, and the span of the pure scion girder becomes larger. Therefore, the implicit condition for the limit span of the hybrid girder to be greater than the limit span of the root and scion girders is *L_r_*$/\sqrt{\gamma }\geq$*L_s_*. Substituting μ = *L_s_*/*L_h_* into Equation (8) gives the following:

(17) $$L_{h}=L_{s}(1-\gamma )+\sqrt{L_{s}^{2}\gamma^{2}-L_{s}^{2}\gamma +L_{r}^{2}}$$

$$\frac{\partial Lh}{\partial Lr}>0,\;\frac{\partial Lh}{\partial Ls}=\left(1-\gamma \right)\left(1-\frac{\gamma Ls}{\sqrt{\gamma \left(\gamma -1\right)L_{S}^{2}+L_{r}^{2}}}\right)$$

$$\frac{\partial Lh}{\partial Ls}\geq 0\Leftrightarrow \frac{L_{r}}{\sqrt{\gamma }}\geq L_{s}$$

Therefore, *L_h_* is a monotonic function of *L_r_* and *L_s_*, and the limit span for hybrid girder is given by Equation (18):

(18) $$L_{hmax}=\sqrt{L_{rmax}^{2}+L_{smax}^{2}\gamma^{2}-L_{smax}^{2}\gamma }+L_{smax}(1-\gamma )$$

**Figure 9:**
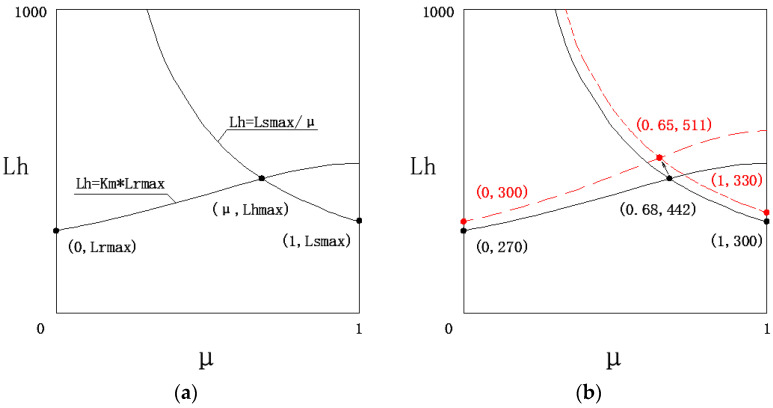
Limit span of hybrid girder bridges: (**a**) the three key factors of limit span; (**b**) improvement of limit span.

Due to construction cost considerations, the rootstock girder in hybrid girder bridges is usually a concrete box girder, while the scion girder can be a corrugated steel web box girder, a steel box girder, or a steel truss girder. Currently, the maximum span for concrete box girders is 270 m (Humen Bridge auxiliary channel bridge), for corrugated steel web box girders is 180 m (Feilong Bridge in Guangxi), for steel box girders is 300 m (Rio-Niterói Bridge in Brazil), and for steel truss girders is 549 m (Quebec Bridge in Canada). The estimated limit spans for various hybrid girders based on the above method are shown in Table 3:

In the three key factors determining the limit span of hybrid girder bridges, *L_rmax_* determines the starting height of the limit span of the hybrid girder, γ (*L_h_* = $\kappa_{M}$ × *L_rmax_* curve) determines the height of the increase, and *L_smax_* (*L_h_* = *L_sma_*_x_/μ curve) determines the upper limit of the increase, ultimately leading to *L_hmax_*, as shown in Figure 9a. Improving any one of these three factors can increase the limit span of hybrid girder bridges. Among them, the most significant factor remains γ. If all three factors are improved simultaneously, the limit span will see a significant increase. Taking the concrete beam–steel box girder hybrid girder bridge as an example, if higher-strength steel is used to reduce the line load intensity ratio γ from 0.3 to 0.25, the maximum span of the concrete box girder bridge increases from 270 m to 300 m, and the maximum span of the steel box girder bridge increases from 300 m to 330 m. As a result, the upgraded concrete beam–steel box girder hybrid girder bridge’s limit span increases from 442 m to 511 m, as shown in Figure 9b.

## 7. Conclusions

Through the study of the equivalent span of hybrid girder bridges, this paper draws the following conclusions:

1. Research on the equivalent span of hybrid girder bridges in this paper is based on hybrid girder components and does not involve factors such as the span-to-span ratio and boundary conditions in system analysis, making it simpler than system analysis. κ, as the core indicator in the equivalent span research, not only represents the mechanical advantage of the hybrid girder components but also serves as the key to the design of the hybrid structure beam bridge system.
2. Through the study of the bending characteristics of uniform hybrid girder components, it was found that the simply supported beam serves as a reliable substitute in the calculation of κ. The Equation (8) based on the simply supported beam model does not rely on the stiffness ratio β but is only related to μ and γ, providing good calculation accuracy. The analysis of the three-dimensional surface and mathematical characteristics of $\kappa_{M}$ derived from Equation (8) enhances engineers’ understanding of hybrid girders.
3. Research on the flexural characteristics of variable cross-section hybrid girders using the simply supported beam model, results in Equation (16). A comparison of five hybrid structure beam bridges with Equation (8) demonstrates that Equation (8) provides sufficient accuracy for conceptual design when empirical values are used. This approach greatly simplifies calculations, highlighting both the simplicity and precision of the equivalent span method.
4. The equivalent span method, verified by fourteen hybrid structure beam bridges, effectively resolves span layout design during the conceptual phase of hybrid structure beam bridges. It determines side-span lengths under fixed hybrid ratios or identifies hybrid ratios for fixed span layouts.
5. By applying Equation (8) in conceptual design, Equation (18) was derived to predict the limit span of hybrid structure beam bridges. This approach is conceptually clear and yields reliable results.
6. The Leq method proposed in this paper also has certain limitations. For example, the hybrid girder component model presented here is based on the mid-span hybrid girder, making it inapplicable to other hybrid girder configurations. Additionally, due to the exclusion of factors such as live load, the calculation accuracy is limited, rendering it suitable only for the conceptual and preliminary design stages of long-span hybrid girder bridges. Addressing these limitations will require further research in the future.

## Figures and Tables

**Table 1:** Comparison of equivalent bending moment span amplification factors for hybrid girders.

Indicators	Stolma Bridge	Shibanpo Bridge	Oujiang Bridge	Anhaiwan Bridge	Taoerhe Bridge
Mid-span length (m)	301	330	200	300	338
End-span length (m)	94	138	84	135	128
Support beam height (m)	15	16	9	15	17
Mid-span beam height (m)	3.5	4.5	3.5	4.5	5.2
Beam height variation curve a	2	1.6	1.6	2	2
Contact face beam height (m)	7.64	6.28	4.77	5.74	8.29
Actual bridge weight ratio g	0.630	0.340	0.365	0.326	0.278
Estimated weight ratio g	0.6	0.3	0.3	0.3	0.3
Hybrid ratio m	0.600	0.312	0.400	0.343	0.512
${\kappa_{M}}_{1}$	1.166	1.185	1.256	1.221	1.388
${\kappa_{M}}_{2}$	1.204	1.238	1.298	1.273	1.491
${\kappa_{M}}_{3}$	1.227	1.259	1.346	1.289	1.464
$(\kappa_{M2}-\kappa_{M1})/\kappa_{M1}$	3.2%	4.5%	3.3%	4.3%	7.4%
$(\kappa_{M3}-\kappa_{M1})/\kappa_{M1}$	5.2%	6.2%	7.1%	5.6%	5.4%

**Table 2:** Span layout of hybrid girder bridges.

Bridges	*L* (m)	g	*m*	$\kappa_{M}$	*L_eq_* (m)	Reasonable Span for End Spans (m)	Actual End Span (m)	Conformance
Stolma Bridge	301	0.6	0.600	1.227	245	123~167	94	×
Shibanpo Bridge	330	0.3	0.312	1.259	262	131~178	138	√
Oujiang Bridge	200	0.3	0.400	1.346	149	74~101	84	√
Anhaiwan Bridge	300	0.3	0.343	1.289	233	116~158	135	√
Ta’erhe Bridge	338	0.3	0.512	1.464	231	115~157	128	√
Cheviré Bridge	242	0.3	0.670	1.630	148	74~101	69.3	×
Zhoushan Bridge	260	0.3	0.327	1.273	204	102~139	125	√
Maogang Bridge	135	0.3	0.407	1.354	100	50~68	65	√
Zhongshan Xiaolan Bridge	220	0.3	0.395	1.341	164	82~112	98	√
Longxiang Bridge	202	0.3	0.396	1.342	151	75~102	93	√
Hangzhou-Wenzhou Railway Bridge	216	0.3	0.380	1.326	163	81~111	100	√
Guangzhan Railway Bridge	200	0.5	0.375	1.199	167	83~113	99	√
Raftsundet Bridge	298	0.6	0.752	1.265	236	118~160	125	√
Niitsu River Bridge, Japan	144	0.7	1.000	1.195	120	60~82	80	√

**Table 3:** Prediction of the limit span of hybrid girder bridges.

Hybrid Girder Bridge (Rootstock Beam Scion Beam)	γ	*L_rmax_*	*L_smax_*	*L_hmax_*	Remarks
Concrete Beam—Corrugated Steel Web Beam	0.70	270	180	311	
Concrete Beam—Steel Box Beam	0.30	270	300	442	
Concrete Beam—Steel Truss Beam	0.25	270	549	540	$L_{r}/\sqrt{\gamma }>L_{s}$, Results are meaningless.

## Data Availability

The original contributions presented in this study are included in the article. Further inquiries can be directed to the corresponding author.

## Notation

*a*	Power function order of the height curve for variable cross-section beams;
*b*	Bending stiffness ratio of the scion girder and the rootstock girder, i.e., (E_s_I_s_)/(E_r_I_r_);
*b*_*1*_	Linear load coefficient related to beam height;
*b*_*1r*_	Linear load coefficient of the rootstock girder related to beam height;
*b*_*1s*_	Linear load coefficient of the scion girder related to beam height;
*b*_*2*_	Linear load coefficient independent of beam height;
*b*_*2r*_	Linear load coefficient of the rootstock girder independent of beam height;
*b*_*sr*_	Linear load coefficient of the scion girder independent of beam height;
*g*	Linear load concentration ratio of the scion girder and the rootstock girder, i.e., qs/qr;
κ	Span increase coefficient;
$\kappa_{M}^{-}$	Equivalent negative bending moment span increase coefficient based on the uniform cross-section fixed-ended beam model;
$\kappa_{M}^{+}$	Equivalent positive bending moment span increase coefficient based on the uniform cross-section fixed-ended beam model;
$\kappa_{M}$	Equivalent bending moment span increase coefficient;
$\kappa_{V}$	Equivalent shear force span increase coefficient;
μ	Hybrid ratio, i.e., scion girder component ratio;
*L*_*eq*_	Equivalent internal force span;
*L*_*h*_	Hybrid girder span;
*L*_*r*_	Rootstock girder span;
*L*_*rmax*_	Maximum span record of rootstock girder;
*L*_*smax*_	Maximum span record of scion girder;

## Appendix A

Details on the derivation of bending moments of the fix-end hybrid beam model.

For the fixed-end hybrid beam model, the structural form and loading conditions are symmetrical, meaning the reaction forces and bending moments at the two boundaries are identical. In the horizontal direction, the corresponding reaction forces are zero, as there are no external horizontal loads acting on the structure. In the vertical direction, the vertical reaction force can be directly calculated using the mechanical equilibrium equation in the vertical direction, given by:

$$F=\frac{q_{r}\left(1-\mu \right)L_{h}+q_{r}\gamma \mu L_{h}}{2}$$

To obtain the bending moments at the two boundaries, the deformation coordination method is applied to the statically indeterminate structure. Let us assume that the bending moments at the two boundaries are *X*_1_ and *X*_2_, respectively. As previously analyzed, *X*_1_ = *X*_2_. Therefore, the displacement coordination condition can be established as follows:

$$\delta_{11}X_{1}=\Delta_{1P}$$

where the coefficients $\delta_{11}$ and $\Delta_{1P}$ can be obtained using the moment-area method, given by

$$\delta_{11}=\int_{0}^{\frac{\left(1-\mu \right)L_{h}}{2}}\frac{M_{1}M_{1}}{EI}dx+\int_{\frac{\left(1-\mu \right)L_{h}}{2}}^{\frac{\left(1+\mu \right)L_{h}}{2}}\frac{M_{1}M_{1}}{\beta EI}dx+\int_{\frac{\left(1+\mu \right)L_{h}}{2}}^{L_{h}}\frac{M_{1}M_{1}}{EI}dx=\frac{\mu L}{\beta EI}-\frac{\left(\mu -1\right)L}{EI}$$

$$\begin{array}{c} \Delta_{1P}=\int_{0}^{\frac{\left(1-\mu \right)L_{h}}{2}}\frac{M_{1}M_{P1}}{EI}dx+\int_{\frac{\left(1-\mu \right)L_{h}}{2}}^{\frac{\left(1+\mu \right)L_{h}}{2}}\frac{M_{1}M_{P2}}{\beta EI}dx+\int_{\frac{\left(1+\mu \right)L_{h}}{2}}^{L_{h}}\frac{M_{1}M_{P3}}{EI}dx\\ =\frac{qL^{3}{\left(\mu -1\right)}^{2}\left(3\gamma \mu -2\mu +2\right)}{24EI}+\frac{qL^{3}\mu \left(6\gamma \mu -6\mu -4\gamma \mu^{2}+3\mu^{2}+3\right)}{24\beta EI} \end{array}$$

where *M*_1_ = 1, and the three coefficients *M*_P1_, *M*_P2_, and *M*_P3_ can be obtained based on the position, given by

$$\left\{\begin{array}{cc} M_{P1}=\frac{q_{r}\left(1-\mu \right)L_{h}+q_{r}\gamma \mu L_{h}}{2}x-\frac{q_{r}x^{2}}{2},\;\; & x\in \left(0,\frac{\left(1-\mu \right)L_{h}}{2}\right)\\ M_{P2}=\frac{q_{r}\left(1-\mu \right)L_{h}+q_{r}\gamma \mu L_{h}}{2}x-\frac{q_{r}\left(1-\mu \right)L_{h}}{2}\left(\begin{array}{c} x-\\ \frac{\left(1-\mu \right)L_{h}}{4} \end{array}\right)-\frac{q_{r}\gamma }{2}{\left(x-\frac{\left(1-\mu \right)L_{h}}{2}\right)}^{2},\; & x\in \left(\frac{\left(1-\mu \right)L_{h}}{2},\frac{\left(1+\mu \right)L_{h}}{2}\right)\\ M_{P3}=\frac{\begin{array}{c} q_{r}\left(1-\mu \right)L_{h}\\ +q_{r}\gamma \mu L_{h} \end{array}}{2}x-\frac{q_{r}\left(1-\mu \right)L_{h}}{2}\left(\begin{array}{c} x-\\ \frac{\left(1-\mu \right)L_{h}}{4} \end{array}\right)-q_{r}\gamma \mu L_{h}\left(\begin{array}{c} x-\\ \frac{L_{h}}{2} \end{array}\right)-\frac{q_{r}}{2}{\left(\begin{array}{c} x-\\ \frac{\left(1-\mu \right)L_{h}}{2} \end{array}\right)}^{2},\; & x\in \left(\frac{\left(1+\mu \right)L_{h}}{2},L_{h}\right) \end{array}\right.$$

Therefore, the bending moments at the two boundaries are calculated as follows:

$$M_{h1}=X_{1}=X_{2}=\frac{\Delta_{1P}}{\delta_{11}}=-\frac{3\beta \gamma \mu (1-\mu {)}^{2}+2\beta {\left(1-\mu \right)}^{3}+(6-4\mu )\gamma \mu^{2}+3\mu (1-\mu {)}^{2}}{24\left[\beta \left(1-\mu \right)+\mu \right]}q_{r}L_{h}^{2}$$

Furthermore, the midspan bending moment can be calculated as follows:

$$M_{h2}=M_{P2}(x=0.5L_{h})+X_{1}=\frac{3\beta \gamma \mu (1-\mu )+\beta (1-\mu {)}^{3}+\gamma \mu^{3}}{24\left[\beta \left(1-\mu \right)+\mu \right]}q_{r}L_{h}^{2}$$

The correctness of the above derivations can be easily validated by setting μ = 0 or μ = 1, which correspond to the conditions of a fully rootstock beam or a fully scion beam, respectively.

## Appendix B

$\kappa_{M}=f\left(\mu ,\gamma \right)$ parameter influence analysis

$$\kappa_{M}=f\left(\mu ,\gamma \right)=\sqrt{\frac{1}{{\left(1-\mu \right)}^{2}+\left(2\mu -\mu^{2}\right)\gamma }}=\sqrt{\frac{1}{\left(1-\gamma \right){\left(1-\mu \right)}^{2}+\gamma }}$$

Domain: $\mu \in \left(0,1\right),\gamma \in \left(0,1\right)$

(1) When γ is constant, the influence of parameter μ on $\kappa_{M}$ is studied.

Monotonicity: $\frac{\partial \kappa_{M}}{\partial \mu }=\left(1-\gamma \right)\left(1-\mu \right){\left[\left(1-\gamma \right){\left(1-\mu \right)}^{2}+\gamma \right]}^{-\frac{3}{2}}>0$, monotonically increasing over the domain.

Convex–concave property: $\frac{\partial^{2}\kappa_{M}}{\partial \mu^{2}}=\left(1-\gamma \right){\left[\left(1-\gamma \right){\left(1-\mu \right)}^{2}+\gamma \right]}^{-\frac{5}{2}}\left[2\left(1+\gamma \right){\left(1-\mu \right)}^{2}-\gamma \right]$ The first two terms are both greater than 0; the inflection point occurs at $\left[2\left(1+\gamma \right){\left(1-\mu \right)}^{2}-\gamma \right]=0$.

i.e., $\mu =1\pm \sqrt{\frac{\gamma }{2\left(1+\gamma \right)}}$, due to $\mu \in \left(0,1\right)$, neglecting the positive

The optimal mixing ratio is $\mu =1-\sqrt{\frac{\gamma }{2\left(1+\gamma \right)}}$, at this point $\kappa_{M}=\sqrt{\frac{2\gamma +2}{\gamma^{2}+3\gamma }}$.

When $\mu <1-\sqrt{\frac{\gamma }{2\left(1+\gamma \right)}}$, $\frac{\partial^{2}\kappa_{M}}{\partial \mu^{2}}>0$; concave curve.

When $\mu >1-\sqrt{\frac{\gamma }{2\left(1+\gamma \right)}}$, $\frac{\partial^{2}\kappa_{M}}{\partial \mu^{2}}<0$; convex curve.

∴ when γ is constant, $\kappa_{M}\left(\mu \right)$ the curve has its endpoints at $\left(0,1\right),\left(1,\frac{1}{\sqrt{\gamma }}\right)$, a monotonically increasing curve that is concave first and then convex, as shown in Figure 6b. Its inflection point between concavity and convexity is $\left(1-\sqrt{\frac{\gamma }{2\gamma +2}},\sqrt{\frac{2\gamma +2}{\gamma^{2}+3\gamma }}\right)$.

The cost of scion girders is generally higher than that of rootstock girders. Therefore, from an economic perspective, a higher mixing ratio is not necessarily better. The $\kappa_{M}\left(\mu \right)$ curve from left to right can be viewed as a dynamic process in which the mid-span section of the rootstock girder is progressively replaced by the scion girder. At the inflection point, the rate of increase in $\kappa_{M}$ reaches its maximum. We define the mixing ratio at this point as the optimal mixing ratio $\mu_{op}\left(\gamma \right)$.

Next, study the relationship between the optimal mixing ratio $\mu_{op}\left(\gamma \right)$ ($\mu_{op}=1-\sqrt{\frac{\gamma }{2\left(1+\gamma \right)}}$) and the parameter γ:

Monotonicity: $\frac{d\mu_{op}}{d\gamma }=-\gamma^{-2}{\left(2+\frac{2}{\gamma }\right)}^{-\frac{3}{2}}<0$, monotonically decreasing within the domain.

Convex–concave property: $\frac{d^{2}\mu_{op}}{d\gamma^{2}}={\left(2+\frac{2}{\gamma }\right)}^{-\frac{5}{2}}\gamma^{-4}\left(4\gamma +1\right)>0$, concave curve

∴$\mu_{op}\left(\gamma \right)$ is a monotonically decreasing concave curve with endpoints at $\left(0,1\right),\left(1,0.5\right)$, as shown in Figure 6c.

(2) When μ is constant, the influence of parameter γ on $\kappa_{M}$ is studied.

Monotonicity: $\frac{\partial \kappa_{M}}{\partial \gamma }=\frac{1}{2}{\left[{\left(1-\mu \right)}^{2}+\left(3\mu -\mu^{2}\right)\gamma \right]}^{-\frac{3}{2}}\mu \left(\mu -2\right)<0$, monotonically decreasing within the domain.

Convex–concave property: $\frac{\partial^{2}\kappa_{M}}{\partial \gamma^{2}}=\frac{3}{4}{\left[\mu \left(\mu -2\right)\right]}^{2}{\left[{\left(1-\mu \right)}^{2}+\left(2\mu -\mu^{2}\right)\gamma \right]}^{-\frac{5}{2}}>0$, concave curve

∴$\kappa_{M}\left(\gamma \right)$ is a monotonically decreasing concave curve, with endpoints at $\left(0,\frac{1}{1-\mu }\right),\left(1,1\right)$, as shown in Figure 6d.
